# Gene function prediction in five model eukaryotes exclusively based on gene relative location through machine learning

**DOI:** 10.1038/s41598-022-15329-w

**Published:** 2022-07-08

**Authors:** Flavio Pazos Obregón, Diego Silvera, Pablo Soto, Patricio Yankilevich, Gustavo Guerberoff, Rafael Cantera

**Affiliations:** 1 Departamento de Biología del Neurodesarrollo, Instituto de Investigaciones Biológicas Clemente Estable, Av. Italia 3318, 11600 Montevideo, Uruguay; 2 Unidad de Bioquímica y Proteómica Analíticas, Instituto Pasteur de Montevideo, Montevideo, Uruguay; 3 Instituto de Investigación en Biomedicina de Buenos Aires (IBioBA), CONICET-Partner Institute of the Max Planck Society, Buenos Aires, Argentina; 4 Instituto de Matemática y Estadística “Prof. Ing. Rafael Laguardia”, Facultad de Ingeniería, UDELAR, Montevideo, Uruguay

**Keywords:** Bioinformatics, Machine learning, Protein function predictions, Comparative genomics

## Abstract

The function of most genes is unknown. The best results in automated function prediction are obtained with machine learning-based methods that combine multiple data sources, typically sequence derived features, protein structure and interaction data. Even though there is ample evidence showing that a gene’s function is not independent of its location, the few available examples of gene function prediction based on gene location rely on sequence identity between genes of different organisms and are thus subjected to the limitations of the relationship between sequence and function. Here we predict thousands of gene functions in five model eukaryotes (*Saccharomyces cerevisiae, Caenorhabditis elegans, Drosophila melanogaster, Mus musculus* and *Homo sapiens*) using machine learning models exclusively trained with features derived from the location of genes in the genomes to which they belong. Our aim was not to obtain the best performing method to automated function prediction but to explore the extent to which a gene's location can predict its function in eukaryotes. We found that our models outperform BLAST when predicting terms from Biological Process and Cellular Component Ontologies, showing that, at least in some cases, gene location alone can be more useful than sequence to infer gene function.

## Introduction

We witness a growing gap between the number of assembled genomes and the number of genes with known functions. Less than 1% of the protein sequences in UniProtKB^1^ have an experimental Gene Ontology annotation^2^ and even in well studied organisms, the majority of known genes have yet no assigned function^3^. Furthermore, well studied genes have frequently been assigned more than one function, so less studied genes, for which only one function is known, have probably more functions to be discovered^4^. In this context there is an increasing need to improve automated function prediction (AFP)^5–9^.

The Critical Assessment of protein Function Annotation algorithms (CAFA) is a series of experiments designed to provide a large-scale assessment of computational methods dedicated to automated function prediction (AFP)^7,10,11^. In all CAFA editions so far, the best results were obtained with machine learning-based methods and combining multiple data sources, typically including sequence derived features, protein structure and molecular interaction data. The performance of the methods evaluated by the CAFA challenges improved dramatically between the first (2013) and the second (2016) edition, but this improvement slowed down between the second and the third edition (2019). The authors hypothesized that including more varied sources of data will lead to additional large improvements in AFP^7^.

Thus, finding new ways to extract relevant biological information from the available data is key to improve AFP. For around 99% of all known proteins, the only available information is the sequence encoded in the corresponding genome, highlighting the importance of sequence-based AFP^12^. But AFP based on sequence similarity is hindered by a highly variable correlation between sequence identity and gene function^13^ and by the evolutionary distance of many genomes to the closest well-characterized genome^14^. Here we explore the hypothesis that the location of a gene relative to other annotated genes of the same genome, a feature that is independent of sequence homology and that can be directly extracted from any annotated genome, is sufficient to perform AFP on eukaryotic genomes, with a performance similar to that reached by sequence similarity alone.

Functionally related genes may be constrained to remain close to each other due to natural selection, forming conserved gene clusters^15^. Local clusters of co-expressed, co-regulated or functionally related genes have been documented in a wide range of organisms, including prokaryotes, yeast, insects, vertebrates and plants^16–23^.

Equating conserved co-locality with co-functionality have been a fruitful approach for the prediction of gene function in prokaryotes for more than 20 years^15,24–28^. On the contrary, there are very few examples^14,29^ of the use of this approach in eukaryotic organisms, although also gene functions are non-randomly distributed in their genomes^21^. However, these AFP studies were based on conserved gene neighborhoods, thus subjected to the limitations mentioned above regarding the relationship between sequence and function.

Here we performed AFP on eukaryotic genomes based exclusively on the relative location of genes. In particular, we tested the predictive power of a feature which represents the spatial organization of genes with respect to their annotated functions, which we term "functional landscape arrays" (FLAs). A FLA is an array associated to each gene, that contains the enrichment in a set of Gene Ontology terms (GO terms) found around the gene, considering different window sizes. These arrays contain information which is independent of sequence similarity between genes and that can be automatically extracted from any annotated genome.

We predicted associations between genes of five well-annotated eukaryote genomes (*Saccharomyces cerevisiae, Caenorhabditis elegans, Drosophila melanogaster, Mus musculus* and *Homo sapiens*) and terms from the three ontologies of Gene Ontology (Biological Process, Cellular Component and Molecular Function) training a set of hierarchical multi-label classifiers with FLAs. Then we compared the results of our 15 models, one for each pair organism/ontology, with equivalent models that randomly assign functions to genes. We found that our models, trained exclusively with location-derived features, performed better than chance in the five organisms and in the three ontologies, showing that there is useful information in the way in which genes are distributed along these genomes.

We also compared the performance of our models to the performance of BLAST, one of the baseline methods of CAFA 3^7^. Using the same approach of the CAFA competitions, we used the updated annotations, released in September 2021, to evaluate the models that we had trained with the annotations released on November 2018. Our models outperformed BLAST when predicting terms form the Biological Process ontology in the three organisms for which specific data from the last CAFA is available and when predicting terms from the Cellular Component ontology our models also performed better in two of these organisms. These results demonstrate that gene location can be informative when performing AFP on eukaryotes. The results also support the idea that gene distribution patterns are tightly regulated in eukaryotic genomes. Finally, our results show that the use of FLAs as predictive feature could complement the annotation of partially annotated genomes.

## Methods

### General procedure to predict associations between genes and GO terms

For each genome,

- Model the genome as a string of protein coding genes.
- Random split in sets T and E, containing 80% and 20% of the genes respectively.

For each Ontology,

- Train a binary classifier for each GO term X associated with at least 40 genes in T and 10 genes in E
- Training set: genes in T annotated with GO term X (as positives) and its siblings (as negatives)
- Predictive feature: a FLA for each gene, including enrichment in GO term X, its siblings and its ancestors
- Hyper-parameters set by grid search & cross validation
- Combine all the binary classifications into one hierarchical multi-label classifier using the node interaction method
- Evaluate performance calculating the hF1 score over the test set E
- Using the classification threshold that maximizes the ratio between the hF1 of the trained model and the hF1 of the random model, predict new associations between GO terms and all the genes in E.

### Genome modeling

We modeled the genome as a collection of segments (the chromosomal arms) in which the protein coding genes -the only elements we considered- are located one next to the other, without intergenic regions or superpositions^30^. In this model, the position of a gene is defined by the location of its transcription starting point and the distance between two genes is the number of other genes located between them. The number of protein-coding genes considered in each genome is shown in Table 1.

**Table 1:** GO terms for which a binary classifier was trained and tested.

Organism	Proetin coding genes	Ontology	Total GO terms	Considered GO terms	hPrec	hRec	hF-max
*S. cerevisiae* (R64)	5892	BP	5074	525	0.24	0.23	0.24
		CC	1035	137	0.51	0.52	0.52
		MF	2323	137	0.69	0.19	0.30
*C. elegans* (WBcel235)	7356	BP	5661	551	0.09	0.15	0.11
		CC	1110	117	0.19	0.33	0.25
		MF	2226	151	0.25	0.14	0.17
*D. melanogaster* (BDGP6)	11,122	BP	7416	880	0.17	0.20	0.18
		CC	1277	176	0.41	0.37	0.39
		MF	2599	212	0.47	0.22	0.30
*M. musculus* (GRCm38.p6)	20,809	BP	15,318	1040	0.22	0.21	0.21
		CC	1953	285	0.46	0.42	0.44
		MF	4269	364	0.63	0.25	0.36
*H. sapiens* (GRCh38.p13)	17,276	BP	13,816	1212	0.21	0.20	0.20
		CC	1818	338	0.44	0.42	0.43
		MF	4244	369	0.47	0.27	0.35

The first column shows the assembly version used for each organism, the second column shows the number of protein coding genes in each genome, the third column indicates the ontology, the fourth column shows the number of GO terms associated with at least one gene for that organism and ontology and the fifth column shows the number of GO terms associated with at least 40 genes in the set T (used for training) and 10 genes in the set E (used for evaluation). These are the GO terms for which a binary classifier was trained and tested. For each organism and ontology, we implemented a hierarchical multilabel classifier combining these binary classifiers. Columns six, seven and eight show the hierarchical precision, recall and F-max reached by each of these models respectively.

### Gene ontology

Gene Ontology (GO) is an attempt to describe all the knowledge about the biological function of genes with three ontologies: Molecular Function, Cellular Component and Biological Process, each one representing different aspects of the biology of a gene product and organized as a directed acyclic graph^2^. Each “GO term” is a node of these graphs, with precise definition and relationships with other terms. A GO annotation occurs when an association between a gene product and a GO term is established. To train our models we used a version of the ontology downloaded on November 2018. To fulfill the true path rule^31^, given the annotations of an organism within a given ontology, we up-propagated all the annotations, meaning that if a gene was annotated with a given GO term we associated that gene with all the ancestor terms up to the root of the graph.

### Local enrichment analysis

Enrichment analysis is a method frequently used to determine if a given gene feature is overrepresented in a list of genes^32^. It assesses if the genes of a list associated with a given feature are more frequent than what should be expected in a list of genes of the same size but randomly picked from the same background list.

**Figure 1:**
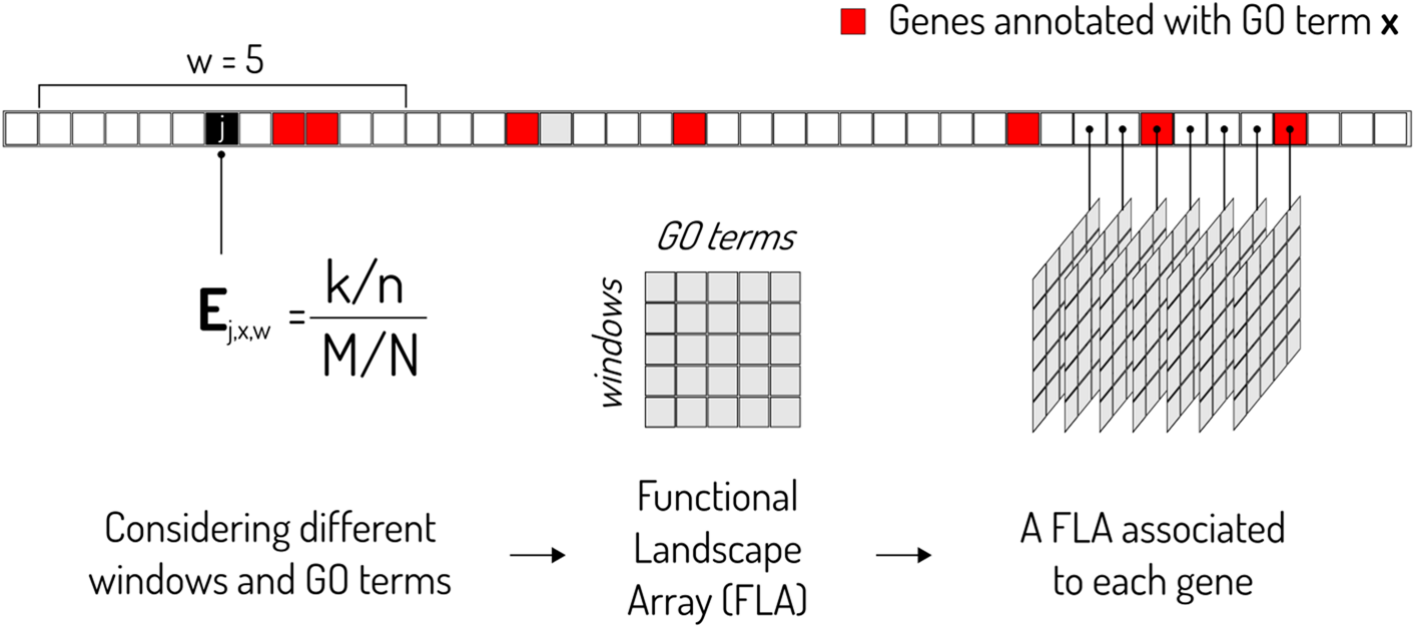
Local enrichment analysis and Functional Landscape Arrays. **k** is the number of genes in the window associated with GO term **x**,** n** is the number of genes in the window, **M** is the number of genes (squares) in the chromosomal arm (strip) associated with GO term **x**, and **N** is the total number of genes in the chromosomal arm.

Given a gene of interest **j**, we define the Local Enrichment in the GO term **x** for the gene **j** and a window **w** centered in **j** as:

1 $${\mathbf{E}}_{{{\mathbf{jxw}}}} = \, \left( {\left( {{\mathbf{k}}/{\mathbf{n}}} \right)/\left( {{\mathbf{M}}/{\mathbf{N}}} \right)} \right)$$

where **N** is the number of genes in the chromosomal arm, **M** is the number of genes in the chromosomal arm associated with GO term **x**, **n** is the number of genes in the window and **k** is the number of genes in the window associated with GO term **x** (see Fig. 1). In other words, **E**_**jxw**_ assess if the genes annotated with the GO term **x** are located in the surroundings of gene **j** more frequently than what could be expected by chance. This approach was successfully used to look for clusters of GO terms along the genome of seven eukaryotes^33^.

### Functional landscape arrays and functional enrichment maps

To functionally characterize the surrounding of a gene we calculated its local enrichment in various GO terms. We considered a window **w**, centered in the gene under consideration, that includes 5, 10, 20, 50 or 100 genes to each side of the gene. The window was moved stepwise one gene at a time until the entire chromosome was covered (see Fig. 1). Then, for each gene we defined a Functional Landscape Array (FLA): an array with a row for each window size and a column for each GO term whose enrichment was evaluated. Because of computational limitations, in the work we are reporting here, the GO terms included in each FLA depend on the GO term to be classified: we only included the enrichment found in that GO term, its father, its siblings and all its descendants.

Importantly: to train our models we did not consider the annotation of the genes in the set E, that was reserved for the evaluation of the models. This procedure guarantees an unbiased evaluation of the classifiers, in which the features used for training are not extracted from examples used for testing. Nevertheless, because it is a useful result by itself, we also performed Local Enrichment Analysis along each genome considering all its current annotations. We calculated the local enrichment around all the genes in each genome using the same set of window sizes and for all those GO terms associated with at least 20 genes and obtained what we call "functional enrichment maps". The functional enrichment map of a given GO term shows which regions of a genome are enriched in that GO term, for various windows sizes.

### Implementation of hierarchical multi label classifiers

We implemented a hierarchical multi label classifier for each pair organism/ontology using, with some modifications, the algorithm proposed in^34,35^. This is a local approach, since a binary classifier is trained for each GO term. Due to computational limitations, for the binary classification at each node, instead of a Support Vector Machine, we used a Random Forest classifier^36^, that has comparable performance in gene function prediction but with lower computational cost. For the same reason we did not use SMOTE^37^, a technique used to artificially generate new labeled data when training sets are too small. Depth, number of trees and measure of impurity for each classifier were set by grid search and threefold cross validation. Supplementary Table 1 includes the hyper parameters of the models.

First, we randomly split the genome into two sets: **T** and **E**. Set **T** included 80% of the genes and was used to define the training sets and to obtain the FLAs. Set **E** included the remaining 20% of the genes and was used to evaluate the models. We trained a binary classifier for each GO term that was associated with at least 40 genes in **T** and at least 10 genes in **E**. Table 1 shows the amount of GO terms meeting these conditions in each organism and ontology, i.e. the GO terms that could be predicted.

To define the training set for each classifier we applied the siblings policy^38^. We included as positive cases those genes associated with the GO term under consideration and as negative cases those genes associated with the siblings or uncles terms of the GO term under consideration and not associated to that term. Importantly, to construct the FLA associated to each gene, to be used as predictive feature, we only considered the annotations of the genes that belonged to **T**.

With each trained classifier we classified the genes in **E** and then post-processed the predictions using the node interaction method^35^, to respect the restrictions imposed by the hierarchy of the ontology. Finally, we evaluated the performance of each hierarchical multi-label classifier using the hierarchical version of the F1 score. All calculations were carried out using ClusterUY (site: https://cluster.uy).

### Evaluation of the models

To evaluate the performance of each trained model we used the complete set of annotations of the genes in **E**, that were not used in training. As evaluation metric we used the hierarchical version of the F1 score (hF1) proposed in^39^ and used in the CAFA competitions. If we denote the true and false positives as TP and FP and the true and false negatives as TN and FN, Precision (Pre) and Recall (Rec) are defined as:

2 $$Pre = {\text{ TP}}/\left( {{\text{TP}} + {\text{FP}}} \right)$$

3 $$Rec = {\text{ TP}}/\left( {{\text{TP}} + {\text{FN}}} \right)$$

and their hierarchical versions, which we term hPre and hRec, are defined as:

4 $$hPrec(\uptheta ) = \frac{{\sum\nolimits_{i = 1}^{n} {\left| {P_{i} (\uptheta ){ \cap }{\rm T}_{i} } \right|} }}{{\sum\nolimits_{i = 1}^{n} {\left| {P_{i} (\uptheta )} \right|} }}$$

5 $$hRec(\uptheta ) = \frac{{\sum\nolimits_{i = 1}^{n} {\left| {P_{i} (\uptheta ){ \cap }{\rm T}_{i} } \right|} }}{{\sum\nolimits_{i = 1}^{n} {\left| {T_{i} } \right|} }}$$

where θ ∈ [0, 1] is the classification threshold, n is the number of genes, T*i* is the set of GO terms truly associated to gene *i* and P*i*(θ) is the set of GO terms predicted for gene *i* with the classification threshold set at θ. We assumed that the root of each ontology always is in P*i*(θ). The hF1 score is the harmonic mean of hPre and the hRec and is defined as:

6 $$hF1(\theta ) = \frac{{2.hPrec(\theta ).h{\text{Re}} c(\theta )}}{{hPrec(\theta ) + h{\text{Re}} c(\theta )}}$$

### Comparison with random models

As a way to assess how far from randomness the distribution of gene functions along the genome is, we compared the hF1 of each of our trained models with the hF1 reached by an equivalent model that assigns the term frequency as the prediction score for any gene. In these "random models", if a given GO term occurs with relative frequency 0.25 in a given genome, the probability of association between each gene of that genome and that GO term is set to 0.25 (Radijovac 2013). For each organism and ontology, we obtained the ratio between the hF1 of the trained model and the hF1 of its random version.

### Comparison to one of the CAFA baseline methods

We also compared the performance of our models to the performance of BLAST, one of the baseline methods used in CAFA 3. In this case, BLAST was based on search results using the Basic Local Alignment Search Tool software against the training database^40^. A term was predicted as the highest local alignment sequence identity among all BLAST hits annotated with the term. BLAST was evaluated during CAFA 3 using the new experimental annotations accumulated during the competition (from February 2017 to November 2017). We used the same approach to evaluate our models, using the annotations files released in September 2021 to evaluate the models that we had trained with the files released on November 2018 .

We compared the performance reached by our models with the performance of BLAST when predicting GO terms for individual species. This data is available as Supplementary files for CAFA 3 at: https://doi.org/10.6084/m9.figshare.8135393.v3 and includes performance evaluation for *H. Sapiens*, *M. musculus* and *D. melanogaster.* We compared our results with those obtained with the limited-knowledge benchmarks and under the full evaluation mode. For more details about the different CAFA evaluations modes please refer to CAFA 3, Additional file 1^7^ and CAFA2^11^.

## Results

### Functional enrichment maps in five model eukaryotes

**Figure 2:**
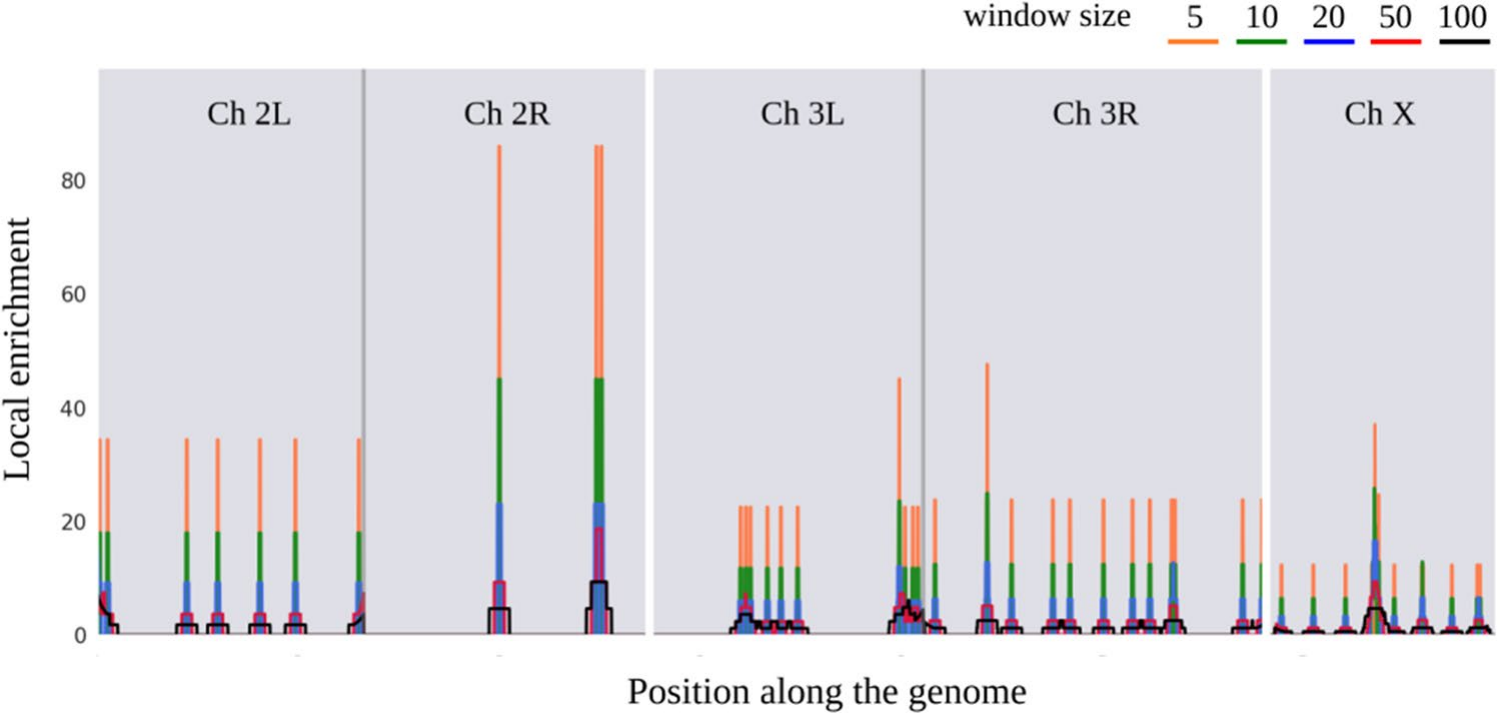
Functional enrichment map of the GO term "Golgi membrane" (GO:0000139) in the genome of *D. melanogaster.* There are 50 *Drosophila* genes annotated with this GO term that belongs to the Cellular Component ontology. The chromosomal position is represented in the x axis and the corresponding local enrichment at each position is shown in the y axis. Each light gray block corresponds to a chromosome (only chromosomes 2, 3 and X are shown) and the vertical dark gray lines mark the position of the centromeres, which divide the chromosome 2 into arms 2L and 2R and chromosome 3 into arms 3L and 3R. The enrichment found using different windows is shown with the colors indicated in the figure.

We performed Local Enrichment Analysis around each gene of a given genome considering windows of various sizes (See “Methods”). Local Enrichment Analysis of a given gene assess if the genes in the surroundings are annotated with any GO term more frequently than what could be expected by chance. Given a GO term, its functional enrichment map shows which regions of a genome are enriched in that GO term, considering various windows sizes. We obtained the functional enrichment map of all those GO terms associated with at least 20 genes in each of the five considered organisms. As an example, Fig. 2 shows the functional enrichment map of the GO term "Golgi membrane" (GO:0000139) in the genome of *D. melanogaster*. The data to generate all the functional enrichment maps is available at: https://github.com/IIBCE-BND/gfpml-datasets/tree/master/lea.

### Implementation of hierarchical multilabel classifiers

We trained fifteen hierarchical multilabel classifiers, one for each possible pair organism/ontology. As detailed in Methods, we randomly split each genome into two sets: **T**, that includes 80% of the genes and was used for training, and **E**, that includes the remaining 20% of the genes and was used for evaluation. Each model assigned probabilities of association between the genes of the set **E** and those GO terms associated with at least 40 genes of the set **T** and 10 genes of the set **E**. Table 1 shows, for each organism and each ontology, the number of GO terms fulfilling these conditions and for which we implemented a binary classifier.

### Evaluation of the models

**Figure 3:**
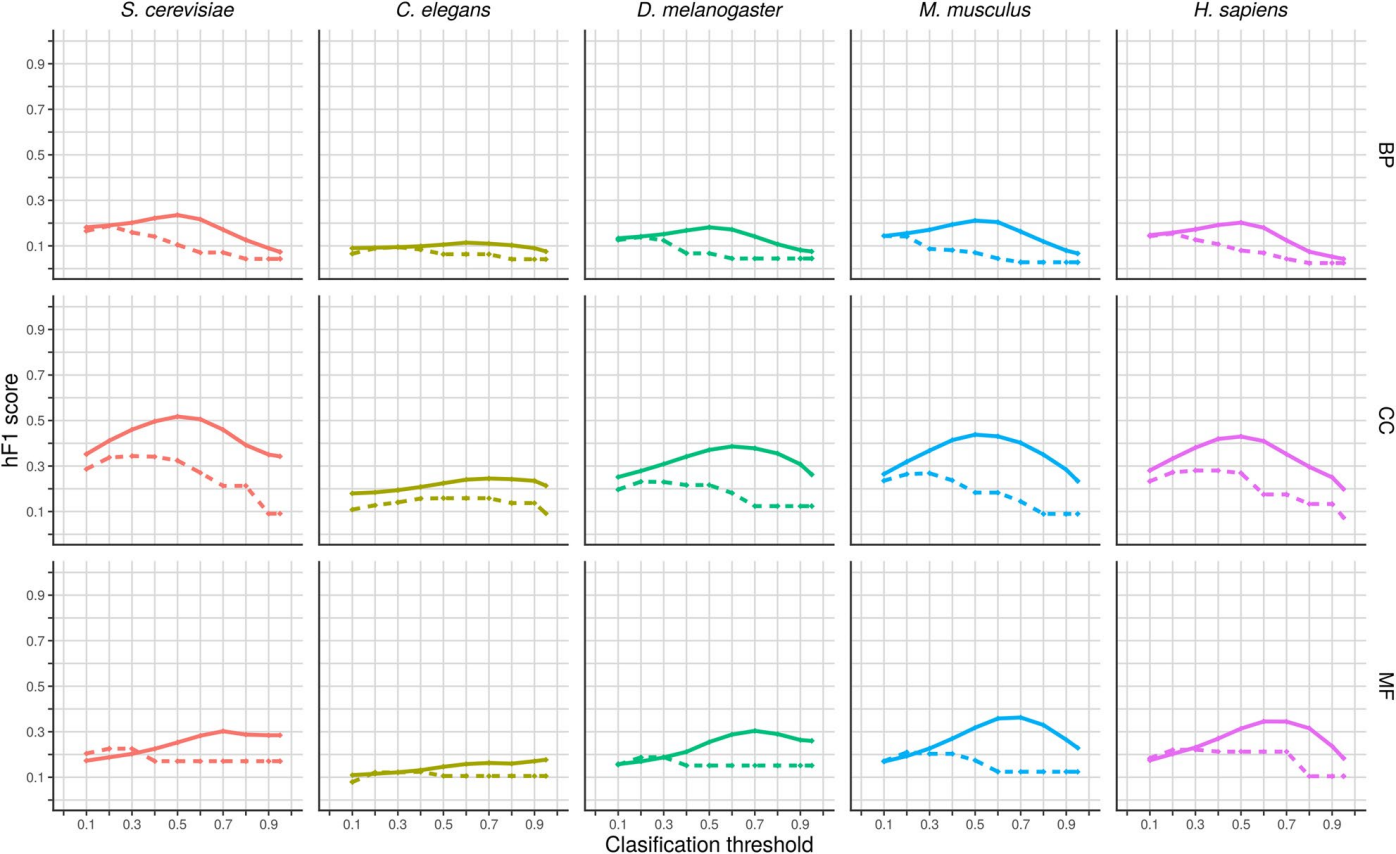
Hierarchical F1 over the test set for each trained and random model as a function of the classification threshold. In each plot the classification threshold, ranging from 0 to 1, is depicted in the x axis and the hF1, also ranging from 0 to 1, is depicted in the y axis. Trained models are represented by solid lines and random models by dotted lines. Each column of the panel corresponds to an organism and each row to an ontology (BP: Biological Process, CC: Cellular Component, MF: Molecular Function).

We evaluated the performance of our models using the hierarchical version of the F1 score (hF1). Figure 3 shows the hF1 reached by each trained model over the test set **E**, as well as the hF1 of the corresponding random model, as a function of the classification threshold.

The hF-max is the highest hF1 score that the model reaches when varying the classification threshold and is a measure of the overall performance of the model. Table 1 shows the hF-max for each model along with the corresponding precision and recall.

### Comparison with random models

**Figure 4:**
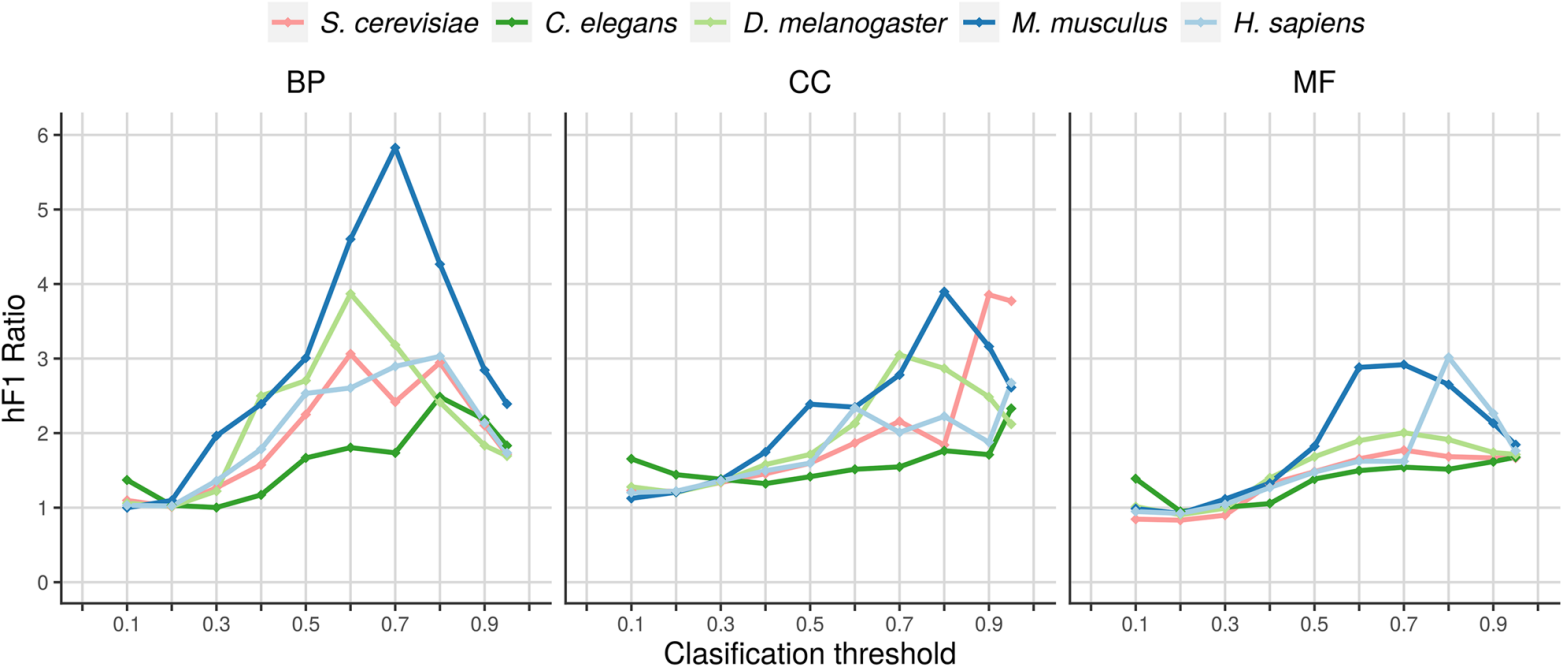
Ratio between the hF1 score of the trained model and the hF1 score of the corresponding random model as a function of the classification threshold. Each graph shows the results for a given ontology, representing each organism with a different color.

**Figure 5:**
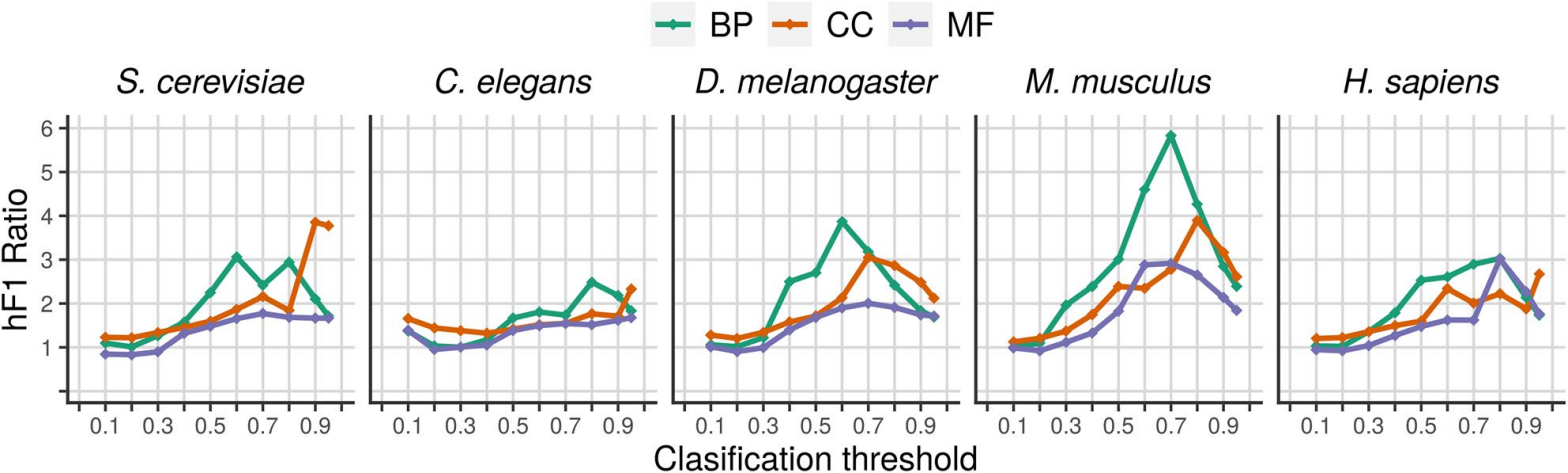
Ratio between the hF1 score of the trained model and the hF1 score of the corresponding random model as a function of the classification threshold. Each graph shows the results for a given organism, representing each ontology with a different color.

To assess how far from randomness the linear organization of the genes along the genome with respect to its functions is, we calculated the ratio between the hF-max of the trained model and the hF-max of an equivalent random model, i.e. a model that assigns the term frequency as the prediction score for any gene (see “Methods”). Figures 4 and 5 show how this ratio varies with the classification threshold in each organism and ontology and Table 2 shows the max ratio between the two models for each pair organism/ontology. The trained models consistently performed better than the random models.

**Table 2:** Max ratio between the hF1 reached by the trained model and the corresponding hF1 reached by the random model over the set E for each possible pair organism/ontology.

Organism	Ontology	Threshold	Max ratio
*S. cerevisiae*	BP	0.60	3.06
	CC	0.90	3.86
	MF	0.70	1.77
*C. elegans*	BP	0.80	2.49
	CC	0.95	2.33
	MF	0.95	1.68
*D. melanogaster*	BP	0.60	3.87
	CC	0.70	3.05
	MF	0.70	2.01
*M. musculus*	BP	0.70	5.83
	CC	0.80	3.90
	MF	0.70	2.92
*H. sapiens*	BP	0.80	3.03
	CC	0.90	2.67
	MF	0.80	3.02

### Comparison to one of the CAFA baseline methods

As a complementary way to evaluate our models, we also compared their performance with the performance reached by BLAST, one of the baseline methods used in CAFA 3 (see “Methods”). “To do so, we used the same approach used during CAFA competitions: we used the annotations released in September 2021 (i.e. after our predictions were generated) to evaluate the performance of the models that we had trained with the files released on November 2018. We compared the hFmax reached by our models with the hFmax reached by BLAST when making predictions for the same individual species (data that is only available for three of the five species we studied here: *H. sapiens, M. musculus* and *D. melanogaster*)"*.*

**Figure 6:**
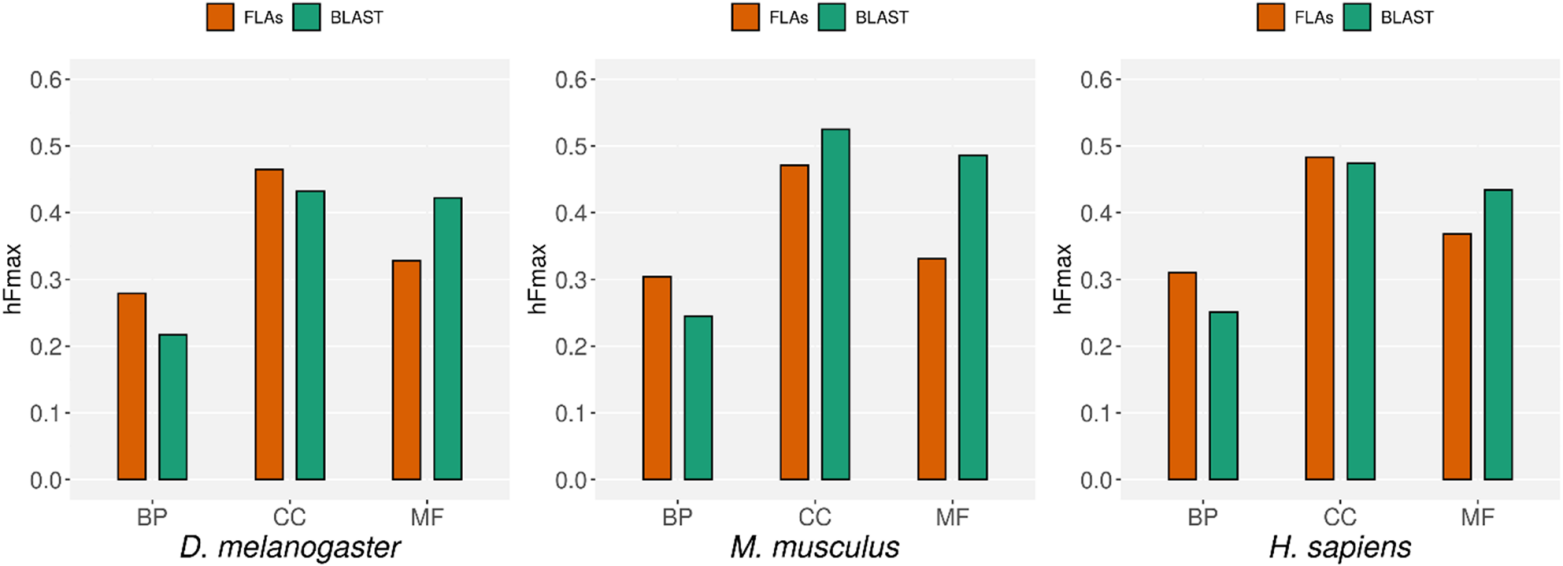
Comparison to one of the CAFA baseline methods. Each graph shows the hFmax of different models when predicting GO terms of the three ontologies in three organisms. In red, the hFmax of the models exclusively trained with FLAs, evaluated using the new experimental annotations accumulated from November 2018 to September 2021. In green, the hFmax of BLAST when making predictions on the same organisms and ontology as reported in CAFA 3^7^.

With this comparison we aimed to asses if gene location alone can predict gene function with a performance comparable to that reached by sequence homology alone. We found that this is the case and Fig. 6 shows the hFmax reached by the three models for each organism and ontology. Notably, for the three considered organisms, the models trained with FLAs outperforms BLAST when predicting GO terms from the Biological Process ontology. Our models also outperform BLAST when predicting GO terms from the Cellular Component ontology in *H. sapiens* and *D. melanogaster.*

### Prediction of new associations between genes and GO terms

**Figure 7:**
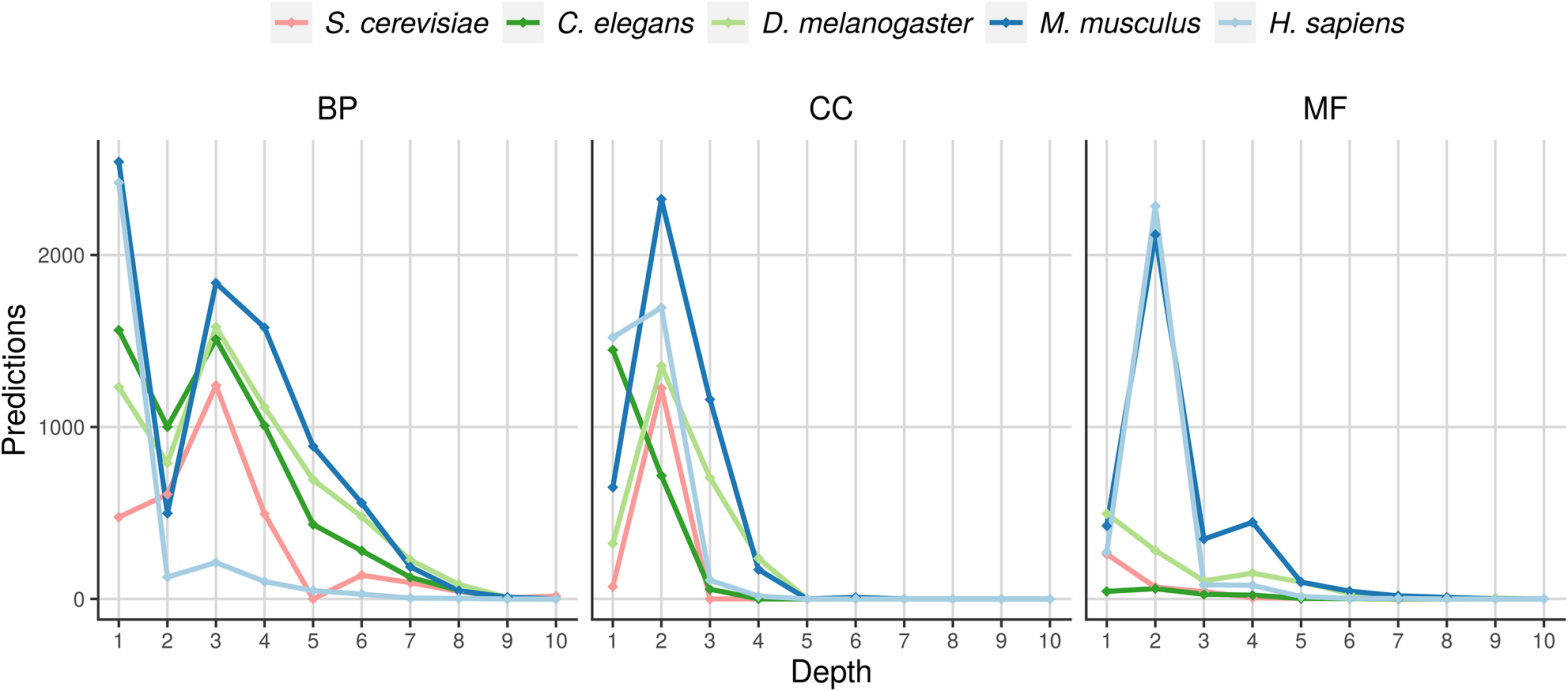
Predictions by depth in the ontology. Each graph corresponds to a different ontology and each organism is shown in a different color. The depth in the ontology is depicted in the x axis and the number of predicted associations above the classification threshold is depicted in the y axis.

In each organism, we classified the genes in the set **E** using the trained model. We obtained the probability of association between each gene in the set **E** and each GO term associated with at least 40 genes in **T** and 10 genes in **E**. We considered as new functional predictions all those associations with probabilities above the classification threshold that maximized the ratio between the hF1 score of the trained model and the hF1 score of the random model. For each gene in the set **E**, we only considered the most specific prediction within a given branch of the ontology. Figure 7 shows, for each ontology and organism, and at each depth of the ontology, the number of new predictions obtained. Because all annotations used for training were up-propagated, along each specific branch of the ontology more general GO terms were always annotated with more genes than more specific GO terms. As our predictions are based on the relative position of existing annotations, along the same branch of the ontology more predictions above the classification threshold should be expected for more general GO terms. The peaks observed in Fig. 7 are a result of the better performance of our method when predicting certain branches of the ontologies.

The complete set of predicted associations with a probability above the threshold is provided as supplementary tables, with one table for each pair organism—ontology (see Supplementary Table S2 to Supplementary Table S16).

## Discussion

For the majority of the known genes, the only available information is their DNA sequence^12^. AFP based on DNA sequence similarity is a common approach, since it is known that two genes with very similar sequences probably have the same function. But the contrary is not always true. A thorough study of the correlation between similarity in protein sequence and function in yeast^13^ found that, although sequence similarity can serve as a key measure in protein function prediction, the majority of the sequences of proteins annotated with the same GO term were non-similar. In general, within one branch of an ontology tree, the more specific a GO term is, the more similar the sequences of the genes annotated with that term are, but the degree of similarity is highly variable and is significant only for specific GO terms. When using orthology between genes, these methods face another limitation: the evolutionary distance of many genomes to the closest well-characterized genome. For example, only 25–50% of the proteins in any given algal genome have detectable sequence similarity to any defined domain in the Pfam database^14^.

The localization of genes along the genome provides an alternative and complementary source of information that is independent of primary sequence^15^. Genomic context-based methods, including gene neighborhoods, gene-order and gene-teams based methods, make use of this information^12^. These methods rely on orthology between genes and thus are subject to the above exposed limitations. Probably because these limitations, the few examples of genomic context-based AFP in eukaryotes are limited to a small proportion of the genes of the organism being considered^29,41^.

There is plenty of evidence pointing to the existence of distinctive patterns in the way in which functionally related genes distribute along eukaryotic genomes. If such patterns are biologically relevant it should be possible, at least in some cases, to predict the functions of a gene using as predictive feature its relative position with respect to other genes of known function in the same genome. As far as we know, here we have performed this task for the first time, using a new way to represent the information contained in these patterns: the Functional Landscape Arrays. This feature can be automatically extracted from any annotated genome and does not depend on orthology relations with other organisms.

Our aim was to explore the hypothesis that the functions of a gene can be predicted from its relative position with respect to other already annotated genes. For that reason, we compared the performance of our method with BLAST, one of the base-line methods used in the CAFA competitions^7^ and not with any of the top performing methods of this competition nor with more sensitive methods as Blast2GO^42^, the state of the art for GO-annotation based on sequence. Using FLAs as the only predictive feature we trained a set of hierarchical multilabel classifiers that outperformed BLAST when predicting GO terms from the Biological Process ontology in *H. sapiens*, *M. musculus* and *D. melanogaster* (see Fig. 6). Our models also outperformed BLAST when predicting GO terms from the Cellular Component Ontology in *H. sapiens* and *D. melanogaster*.

Our study resulted in the prediction of thousands of associations between several hundreds of GO terms and thousands of genes from five different organisms. It is thus not feasible to either validate or provide a theoretical justification in our publication for all those genes or even for a representative proportion of them. However, we hope the following examples makes a convincing argument in favor of our predictions:

− MYCT1 encodes a protein predicted to act upstream of or within hematopoietic stem cell homeostasis. Our model predicted the association between MYCT1 and the GO term "regulation of gene expression". Later on, a study published after the date of the annotation files we used to train our models, suggested that MYCT1 synergistically interact with MAX as a co-transcription factor or a component of MAX transcriptional complex, involved in enhanced apoptosis in laryngeal cancer cells^43^. The following year, another study found that MYCT1 significantly decreases the expression of miR-629-3p but increased the expression of ESRP2 in laryngeal cancer cells^44^.

− Tmem132e encodes a transmembrane protein known to be involved in the posterior lateral line neuromast hair cell development. Our model had predicted the association between Tmem132e and the GO term "response to IFN-γ". A study published in 2019 included Tmem132e as one of the top genes dysregulated by Notch1 haploinsufficiency in the presence of LPS/IFN-γ^45^.

All the predictions obtained with our trained classifiers are provided as supplementary tables.

The relevance of our results stems from the fact that the performance of our models, assessed by standard metrics, shows that AFP exclusively based on features derived from the relative location of genes can be successfully performed on eukaryotic genomes. Even though, in AFP, it is common practice to integrate multiple types of information, information derived from gene location is rarely taken into account. Furthermore, according to the CAFA organizers, new improvements in gene function prediction should be expected from the incorporation of new kinds of predictive features^7^. We believe that including FLAs as predictive feature could significantly improve the performance of AFP models.

The use-case of our method is a partially annotated genome. When dealing with a novel genome with predicted genes/gene products, typically the first step is to annotate as many genes as possible based on sequence similarity. But because annotation based on sequence similarity has some drawbacks, a significant part of the genes will remain unannotated. For example, in yeast the majority of the sequences of proteins annotated with the same GO term are non-similar^13^. Moreover, after using all other known sources of information (as phylogeny, interaction networks, etc.) to predict new annotations and after years of experimental work, the genomes of the most studied model organisms are still incompletely annotated, with thousands of genes without any annotation. We think the utility of our method is precisely to complement all other known sources of information used to predict gene function and improve annotations.

Our results are interesting from another point of view. The existence in eukaryotes of distribution patterns of functionally related genes so well defined as to allow good AFP points to levels of organization thought to be exclusive of prokaryotic genomes and its characteristic operons^46^. Diament and Tuller performed a comparative study of the organization of several genomes, analyzing the location of functionally related genes. Their results revealed that the prokaryote *Escherichia coli* exhibits a higher level of genomic organization than the eukaryote *S. cerevisiae*, as one would expect given its operon-based genomic organization. But when considering a higher order of genomic organization, analyzing the co-localization of pairs of different functional gene groups, the authors found that the genome of *S. cerevisiae* is markedly more organized than that of *E. coli*. Our results are consistent with this trend.

To estimate how far from randomness the distribution of the annotations corresponding to different ontologies and different genomes is, we used the hF-max ratio, i.e. the ratio between the hF-max reached by the trained model and the hF-max reached by an equivalent random model. Table 2 and Fig. 4 show that although the relationship between the complexity of the organism and its hF-max ratio is not linear, simpler organisms reach lower hF-max ratios than more complex organisms. Figure 5 shows that, for the five considered organisms, hF-max ratio is higher for Molecular Function than for Biological Process, which in turn is higher than the ratio for Cellular Component. This result suggests that gene location has better predictive power over gene function when dealing with the Molecular Function ontology.

In sum, Functional Landscape Arrays have the potential to improve AFP, as they can be easily integrated into any model, can be automatically extracted from any annotated genome and are independent of sequence identity. To the best of our knowledge, this is the first work in which only features derived from the relative gene location of the genes within a genome are used to successfully predict gene function in eukaryotes.

## Supplementary Information

Supplementary Table S1.

Supplementary Table S2.

Supplementary Table S3.

Supplementary Table S4.

Supplementary Table S5.

Supplementary Table S6.

Supplementary Table S7.

Supplementary Table S8.

Supplementary Table S9.

Supplementary Table S10.

Supplementary Table S11.

Supplementary Table S12.

Supplementary Table S13.

Supplementary Table S14.

Supplementary Table S15.

Supplementary Table S16.

## Data Availability

All data generated or analysed during this study are included in this published article (and its Supplementary Information files). The code and data used to train and evaluate the models is available at: https://github.com/IIBCE-BND/gfpml-models*,* https://github.com/IIBCE-BND/gfpml-tools and https://github.com/IIBCE-BND/gfpml-datasets. The data to generate all the functional enrichment maps is available at: https://github.com/IIBCE-BND/gfpml-datasets/tree/master/lea.
